# P-glycoprotein Expression Is Upregulated in a Pre-Clinical Model of Traumatic Brain Injury

**DOI:** 10.1089/neur.2020.0034

**Published:** 2020-11-18

**Authors:** Sydney M. Vita, John B. Redell, Mark E. Maynard, Jing Zhao, Raymond J. Grill, Pramod K. Dash, Bernadette E. Grayson

**Affiliations:** ^1^ Department of Neurobiology and Anatomical Sciences, University of Mississippi Medical Center, Jackson, Mississippi, USA.; ^2^ Department of Neurobiology and Anatomy, University of Texas McGovern Medical School, Houston, Texas, USA.

**Keywords:** blood–brain barrier, hippocampus, P-glycoprotein, traumatic brain injury

## Abstract

Athletes participating in contact sports are at risk for sustaining repeat mild traumatic brain injury (rmTBI). Unfortunately, no pharmacological treatment to lessen the pathophysiology of brain injury has received U.S. Food and Drug Administration (FDA) approval. One hurdle to overcome for potential candidate agents to reach effective therapeutic concentrations in the brain is the blood–brain barrier (BBB). Adenosine triphosphate (ATP)-binding cassette (ABC) transporters, such as P-glycoprotein (Pgp), line the luminal membrane of the brain capillary endothelium facing the vascular space. Although these transporters serve to protect the central nervous system (CNS) from damage by effluxing neurotoxicants before they can reach the brain, they may also limit the accumulation of therapeutic drugs in the brain parenchyma. Thus, increased Pgp expression following brain injury may result in reduced brain availability of therapeutic agents.

We therefore questioned if repeat concussive injury increases Pgp expression in the brain. To answer this question, we used a rodent model of repeat mild closed head injury (rmCHI) and examined the messenger RNA (mRN) and protein expression of both isoforms of rodent Pgp (Abcb1a and Abcb1b). Compared with sham-operated controls (*n =* 5), the mRNA levels of both Abcb1a and Abcb1b were found to be increased in the hippocampus at day 1 (*n =* 5) and at day 5 (*n =* 5) post-injury. Using a validated antibody, we show increased immunolabeling for Pgp in the dorsal cortex at day 5 and in the hippocampus at day 1 (*n =* 5) and at day 5 (*n =* 5) post-injury compared with sham controls (*n =* 6). Taken together, these results suggest that increased expression of Pgp after rmCHI may reduce the brain accumulation of therapeutic drugs that are Pgp substrates. It is plausible that including a Pgp inhibitor with a candidate therapeutic agent may be an effective approach to treat the pathophysiology of rmCHI.

## Introduction

Traumatic brain injury (TBI) is a major health care problem in the United States, with more than 2 million new cases occurring annually.^1^ Brain injury can be classified by severity level (i.e., mild, moderate, or severe) and/or mechanism (e.g., direct impact, penetrating, or acceleration/deceleration).^2,3^ It has been estimated that more than 75% of TBI cases are classified as mild (mTBI), also commonly referred to as concussion.^4,5^ Although most patients report the abatement of symptoms within 3 months, as many as 33% may experience persistent symptoms for months or years post-injury.^6^ The mechanisms through which mTBI alters brain function and pathology at various time-points are not well understood and are an intense area of study.

Currently, there are no effective therapeutics available to lessen TBI pathobiology and improve brain function. One hurdle for candidate agents to overcome is crossing the blood–brain barrier (BBB) to reach the brain. Research into spinal cord injury (SCI) offers a mechanism by which central nervous system (CNS) availability of pharmacological agents can be restricted. SCI creates a condition reminiscent of chemotherapeutic resistance,^7^ a pathological condition first described in tumors that develop resistance to drugs due to upregulation of P-glycoprotein (Pgp). Pgp is a broad specificity adenosine triphosphate (ATP)-dependent efflux pump that can bind to hundreds of different substrates (including therapeutic drugs) and pump them out of the tissue.^8^ As a result, systemically administered drugs may not be able to achieve a concentration in the injured spinal cord sufficient to exert therapeutic effects. This phenomenon has also been shown to occur under other pathological conditions, such as in a mouse model of amyotrophic lateral sclerosis (ALS). It has been demonstrated that Pgp is responsible for limiting the bioavailability of riluzole, a U.S. Food and Drug Administration (FDA)-approved treatment for ALS, and that co-administering riluzole with a Pgp inhibitor improves treatment efficacy.^9,10^

In humans, a single, severe TBI has been reported to acutely increase the expression of Pgp in peri-contusional brain biopsy samples.^11^ Similarly, a significant increase in Pgp expression has been seen at the site of injury 3 days following experimental SCI.^7^ However, it has not been investigated if mTBI, particularly repeat mild TBI (rmTBI), alters Pgp expression. In the current study, we test the hypothesis that rmTBI will elicit an upregulation of Pgp, suggestive of a chemotherapeutic resistant-like state. We chose to evaluate Pgp expression at 1 day and 5 days post-injury as some patients with concussion may seek care acutely after injury, whereas others may delay and only seek care if symptoms worsen, or do not resolve.

## Methods

### Animal assurance

All animal care and experimental procedures were conducted in accordance with the “Guide for the Care and Use of Laboratory Animals” of the National Institutes of Health and approved by the Institutional Animal Care and Use Committee of the University of Texas Health Sciences Center.

### Animals

We utilized male Mdr1a/b^–/–^ constitutive knockout (KO) mice bred on an FVB background (FVB.129P2-Abcb1a^tm1Bor^Abcb1b^tm1Bor^, Taconic Biosciences, Germantown, NY, USA) and wild type (WT) controls for our antibody validation studies. C57BL/6 male mice (12–16 weeks old; Jackson Laboratories, Bar Harbor, ME, USA) were used for all other experiments. All animals were housed singly on a 12-h light/dark cycle with *ad libitum* access to food and water.

### Injury

Repeat, mild closed head injury (rmCHI) was delivered using a pneumatically driven controlled cortical impact (CCI) device, as previously described.^12,13^ C57Bl/6 mice (20–25 g, *n =* 31) were anesthetized initially with 5% isoflurane in a 1:1 O_2_/air mixture, and then maintained on a 2.5% isoflurane and 1:1 O_2_/air mixture via a face mask. Animals were mounted on a stereotaxic frame to provide stability as a midline incision was made on the scalp, and the soft tissue was reflected to expose the skull. The mouse was then transferred to a foam pad designed to hold the head at a level plane with the body. Anesthesia was discontinued for 20 sec (the time required to regain a tail pinch reflex) before a 5-mm diameter metal tip delivered a single impact to the skull. The impact was made over the sagittal suture, midway between lambda and bregma, at a velocity of 5.0 m/sec to a depth of 1.0 mm. After normal breathing was observed, the scalp was closed using sterile surgical staples. Mice received one injury per day for 4 consecutive days. Sham mice received daily isoflurane anesthesia but were not injured. Total number of mice used for experiments is as follows: *n =* 11, sham; *n =* 10, euthanized 1 day following 4 consecutive days of injury (1 day rmCHI); and *n =* 10, euthanized 5 days following 4 consecutive days of injury (5 days rmCHI).

### Euthanasia and tissue processing

#### Western blot analysis

Total protein homogenates were prepared from constitutive Mdr1a/b^–/–^ mice and controls as previously described.^13^ Total protein concentration was measured by bicinchoninic acid (BCA) assay using bovine serum albumin (BSA) as the reference standard. Sample aliquots were diluted into 1 × Wes sample buffer and protein content equalized. Target proteins were quantified using an automated capillary immunoassay system (Wes, Protein Simple, San Jose, CA, USA).

#### Brain harvest for RNA

At 1 day (*n =* 5) and 5 days (*n =* 5) following the final injury, or 1 day (*n =* 3) and 5 days (*n =* 2) following sham procedure, animals were rapidly decapitated, brains were removed, hippocampi were quickly dissected out on ice, and tissues were frozen on dry ice in Eppendorf tubes. Tissues were stored at −80°C until needed for processing.

### RNA isolation and real-time polymerase chain reaction

RNA was extracted using a QIAGEN miniprep RNA kit (QIAGEN, Inc., Valencia, CA, USA), and concentration of total RNA was determined by NanoDrop Lite (Thermo Scientific). Complementary DNA (cDNA) was generated from 1000 ng total RNA using an iScript cDNA synthesis kit (Bio-Rad Laboratories, Hercules, CA, USA). Quantitative polymerase chain reaction (PCR) was performed on a Step-One Plus Real-Time PCR (RT-PCR) instrument running StepOne software (version 2.3, Applied Biosystems) using TaqMan inventoried gene expression assays (Life Technologies, Foster City, CA, USA) as listed in Table 1. Samples were analyzed in duplicate and the change in threshold cycle (C_T_) values from the reference control (L32) was calculated. Values obtained for the control group were averaged and made to equal 100 for presentation purposes.

**Table 1.** Primers for PCR

Name	Gene symbol	Assay ID
Pgp a	Abcb1a	Mm00440761_m1
Pgp b	Abcb1b	Mm00440736_m1
BCRP	Abcg2	Mm00496364_m1
Mrp1	Abcc1	Mm00456156_m1
Mrp4	Abcc4	Mm01226381_m1
Mrp5	Abcc5	Mm01343626_m1
Mrp6	Abcc6	Mm00497698_m1
Claudin 1	Cldn1	Mm01342184_m1
Claudin 5	Cldn5	Mm00727012_s1
Occludin	Ocln	Mm00500912_m1
Zo-1	Tjp1	Mm01320638_m1
L32	Rpl32	Mm02528467_g1

BCRP, breast cancer resistance protein; PCR, polymerase chain reaction; Pgp, P-glycoprotein.

### Immunohistochemistry

At 1 day (*n =* 5) and 5 days (*n =* 5) following the final injury, or 1 day (*n =* 3) and 5 days (*n =* 3) following sham procedure, animals were deeply anesthetized using intraperitoneal sodium pentobarbital (100 mg/kg). Once animals failed to respond to toe and tail pinch, they were transcardially perfused with ice-cold saline followed by decapitation. Brains were removed and flash frozen in −80°C isopentane (2-methylbutane). Cryosections (20 μm thick) were prepared using a cryostat and were directly mounted onto Leica Apex™ Superior Adhesive Slides (Leica Biosystems, Buffalo Grove, IL, USA). Slides were stored at −20°C until needed for processing. Three sections per animal corresponding to approximately bregma 1.80 mm were post-fixed in 100% MeOH methanol for 10 min at −20°C, then allowed to dry. All subsequent steps were completed at room temperature. Tissues were permeabilized for 45 min in phosphate-buffered saline (PBS) +0.25% Triton, then blocked in PBS +5% normal goat serum for 1 h. Primary antibodies were diluted in PBS +2.5% goat serum to a final concentration of 7 μg/mL and were incubated overnight. After extensive washing in PBS, sections were incubated for 2 h in corresponding secondary antibodies conjugated to an Alexa Fluor dye for visualization (final concentration of 2 μg/mL). Finally, sections were washed for 2 min in bisbenzimide (#382061, Calbiochem) diluted to 1 μg/mL in 1 × PBS.

### Immunolabeling analysis

Fluorescent sections were imaged using an Axioscan.Z1 slide scanning microscope (Zeiss, Thornwood, NY, USA) equipped with an X-Cite XLYS LED light source (Excelitas Technologies, Waltham, MA, USA) and custom filter sets (Semrock). Images were captured using a 20 × /0.8 NA Plan-Apochromat objective lens (Zeiss) and ORCA-Flash4.0 sCMOS digital camera (Hamamatsu) at 325 nm/pixel resolution and were montaged using Zen Blue 2.3 software. Exposure and gain were adjusted using a representative section from a sham-operated animal and were kept constant across all groups. Fluorescence intensity signals for each region of interest (ROI) were quantified for each hemisphere and then averaged. Bisbenzimide staining was used as a guide to trace the ROIs in ImageJ. ROIs were identified using a mouse brain atlas.^14^ Off-target fluorescence intensity was measured for each section, averaged, and subtracted from the target signal for quantification. The corrected intensity was then averaged across the three sections for each animal and data (mean ± standard error of the mean [SEM]) presented relative to Sham (set to 100%).

### Antibodies

The following antibodies were used in these studies: anti-Pgp antibodies (#ab170904, #ab3366, AbCam, Cambridge, MA; and MA1-26528, Thermo Scientific), astrocytic marker glial fibrillary acidic protein (GFAP; custom, Bethyl, Montgomery, TX, USA), microglia marker Iba1 (# 234004, Synaptic Systems), tight junction marker occludin (#33-1500, Thermo Scientific), and neuronal marker NeuN (Millipore, # ABN91). Species-specific secondary antibodies linked to Alexa Fluor dyes (Alexa Fluor 488, Alexa Fluor 594, and AlexaFluor 647) were purchased from Thermo Fisher Scientific.

### Statistical analysis

All statistical analyses were performed using GraphPad Prism version 8.3.1 (GraphPad Software, San Diego, California, USA). When testing across time, a one-way analysis of variance (ANOVA) was performed followed by post hoc analysis. Differences between two groups were assessed using an unpaired Student's *t* test with a two-tailed distribution. Results were considered significant at *p* < 0.05, and data are shown as mean ± SEM.

## Results

### rmCHI triggers an inflammatory response but does not cause overt neuronal loss

**FIG. 1.**
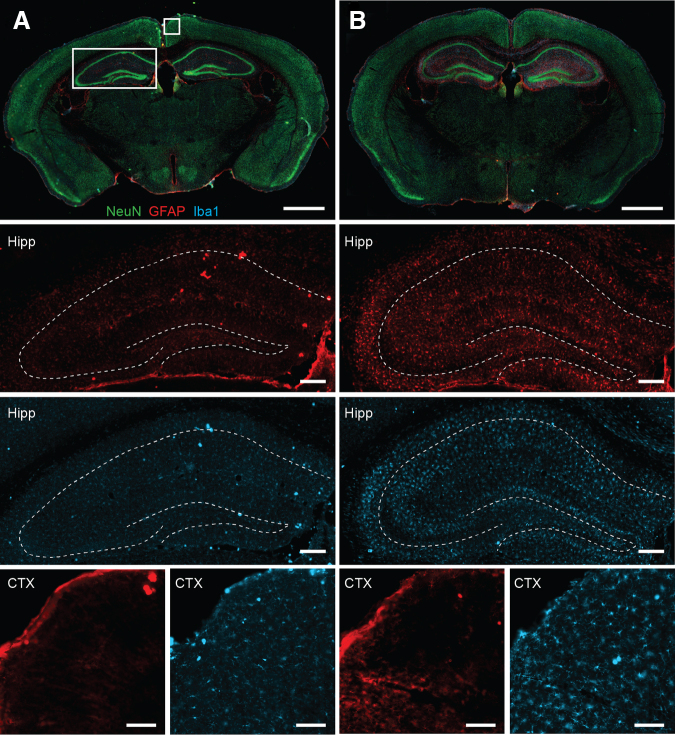
rmCHI triggers an inflammatory state but not overt neuronal cell loss. Representative images of brain sections from **(A)** sham and **(B)** rmCHI animals 24 h after the last injury or sham surgery, stained with the neuronal marker NeuN (green), the astrocyte maker GFAP (red), and the microglial marker Iba-1 (blue). The boxed areas in (**A**) indicate the regions of the cortex and hippocampus that are shown in the higher magnification panels. Dashed lines indicate the position of the CA1/3 and dentate granule cell body layers in the hippocampus. Scale bars: whole brain panel: 1 mm; hippocampus panels: 200 μm; cortex panels: 100 μm. GFAP, glial fibrillary acidic protein; rmCHI, repeat mild closed head injury.

To examine for overt neuronal loss after rmCHI, tissue sections from sham (Fig. 1A) and rmCHI (Fig. 1B) mice were stained for NeuN (a marker of neurons). GFAP immunoreactivity was used to examine the inflammatory response after rmCHI. Representative images from a sham and rmCHI mouse are shown in Figure 1. Visual examination of NeuN signal intensity and localization did not reveal any areas with overt evidence of neuronal cell loss, whereas enhanced GFAP immunoreactivity was readily apparent throughout the hippocampus and overlying corpus callosum, in addition to some increased staining in the cortex, as we and others have previously observed.^12,13,15,16^ Increased immunoreactivity of the microglial marker Iba-1 was also observed in the hippocampus, overlying white matter and cortex.

### rmCHI increases Pgp mRNA expression in the hippocampus

**FIG. 2.**
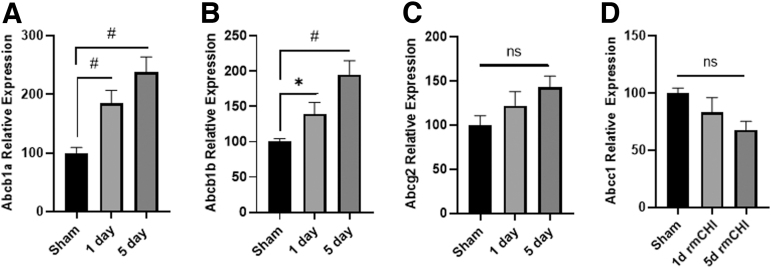
RT-PCR of ABC transport proteins in Sham, 1 day post-rmCHI, and 5 days post-rmCHI. Expression shown as normalized to L32. (**A)** Abcb1a; (**B)** Abcb1b; (**C)** Abcg2; (**D)** Abcc1. Overall effect is shown. *denotes *p* < 0.05; #denotes *p* < 0.01. ABC, ATP-binding cassette; rmCHI, repeat mild closed head injury; RT-PCR, real-time polymerase chain reaction.

Pgp is a member of the ABC family of transport proteins, with two isoforms (Abcb1a and Abcb1b) expressed in rodents. To examine if rmCHI alters Pgp messenger RNA (mRNA) expression, brain tissues were collected 1 or 5 days after the last injury, and total RNA was extracted and used for quantitative RT-PCR analysis using isoform-specific primers (Table 1). Ribosomal RNA L32 was used as an internal reference for sample to sample normalization. Results presented in Figure 2 show the time course for mRNA changes in Abcb1a and Abcb1b mRNA levels in the hippocampus, a structure critical for learning and memory that is vulnerable to TBI. Abcb1a mRNA in the hippocampus was significantly increased in rmCHI animals as compared with sham (one-way ANOVA, F_(2,12)_ = 11.50) by 1 day post-injury (*p* < 0.01), and was further increased to greater than twofold by 5 days post-injury (*p* < 0.01; Fig. 2A). Abcb1b mRNA showed a similar response after rmCHI (one-way ANOVA, F_(2,12)_ = 10.39), with a significant increase observed by 1 day post-injury (*p* < 0.05), and a greater response observed by 5 days post-injury (*p* < 0.01; Fig. 2B). To determine if this might be a generalized response to traumatic injury, we next assessed the mRNA expression levels of two related ABC transporter superfamily members, Abcg2 and Abcc1. Abcg2, also known as breast cancer resistance protein, or BCRP, showed a non-significant upward trend in mRNA expression (F_(2,12)_ = 3.10; *p* = 0.085; Fig. 2C), whereas Abcc1 exhibited the opposite (F_(2,12)_ = 2.76; *p* = 0.107; Fig. 2D).

### Authentication of Pgp antibodies

We attempted to assay Pgp expression in hippocampal and cortical homogenates using a commercially available enzyme-linked immunosorbent assay (ELISA) kit (#LS-F12021-1, LSBio, Seattle, WA, USA). Although the standard curve that was generated using reagents supplied with the assay showed a linear increase in signal with increasing protein concentration, we found the results obtained using brain tissue extracts were highly variable. We therefore questioned if antibody specificity might contribute to the variability we observed. To address this, Mdr1a/1b^–/–^ constitutive dual KO mice (with *Mdr1a* exons 3–4 disrupted with a hygromycin targeting cassette, and *Mdr1b* exons 3–4 disrupted by a neomycin targeting cassette^17^) were purchased, and brain extracts prepared for ELISA and western blot analysis. Brains from WT FVB mice were used as a control. Equal amounts of KO and WT brain homogenate were tested by ELISA, and a strong immunoreaction was detected in Mdr1a/b^–/–^ mice (Supplementary Fig. S1A). Similarly, when extracts from WT and KO mice were run in a capillary western blot, immunoreactive bands were detected in both brain tissues, suggesting that the anti-Mdr1 antibody supplied with the kit may be non-specific (Supplementary Fig. S1B).

**FIG. 3.**
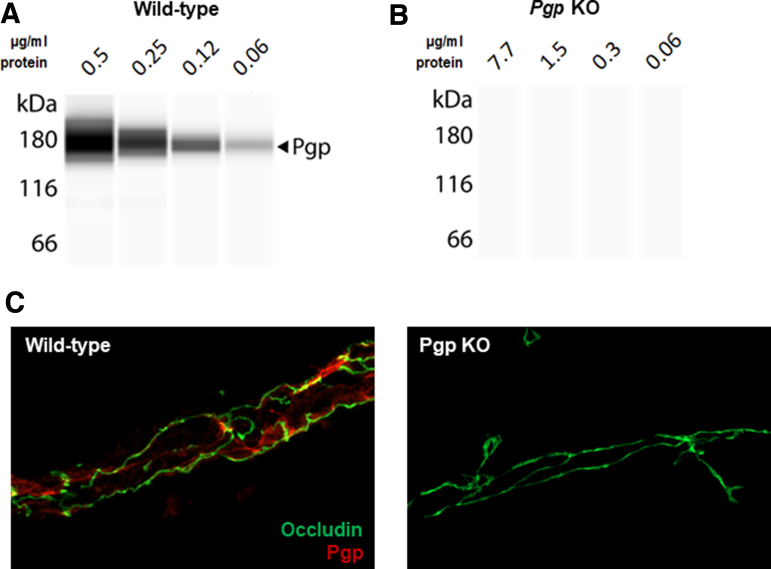
Validation of AbCam anti-Pgp antibody (#ab170904). **(A)** Antibody validation in wild-type animals via Wes. **(B)** Antibody validation in KO animals via Wes. **(C)** Antibody validation via confocal imaging in wild-type and KO animals. Overall effect is shown. *denotes *p* < 0.05; #denotes *p* < 0.01. KO, knockout; Pgp, P-glycoprotein.

We therefore screened three different commercially available Pgp antibodies and verified one (Abcam #ab170904) as being selective for Pgp in western blot and immunohistochemistry. Figure 3A shows a representative capillary western blot using this antibody indicating the linearity of Pgp immunoreactivity in relationship to different amounts of starting material (WT cortical extract). The 180 kDa band was not observed in brain extracts from Mdr1a/b^–/–^ mice (Fig. 3B). We then stained WT and Mdr1a/1b^-/-^ brain sections for occludin (tight junction protein expressed on microvessels) and Pgp (#ab170904). Figure 3C shows that Pgp and occludin co-labeled microvessels in the WT brain, but Pgp immunoreactivity was absent in microvessels in the KO brain sections. Although several studies have used the mouse monoclonal antibody (C219; #MA1-26528),^18–22^ the rabbit monoclonal antibody (#ab170904) provided the most robust western blot and immunohistochemistry signals in our hands.

### rmCHI is associated with increased Pgp immunoreactivity in the dorsal cortex and the hippocampus

**FIG. 4.**
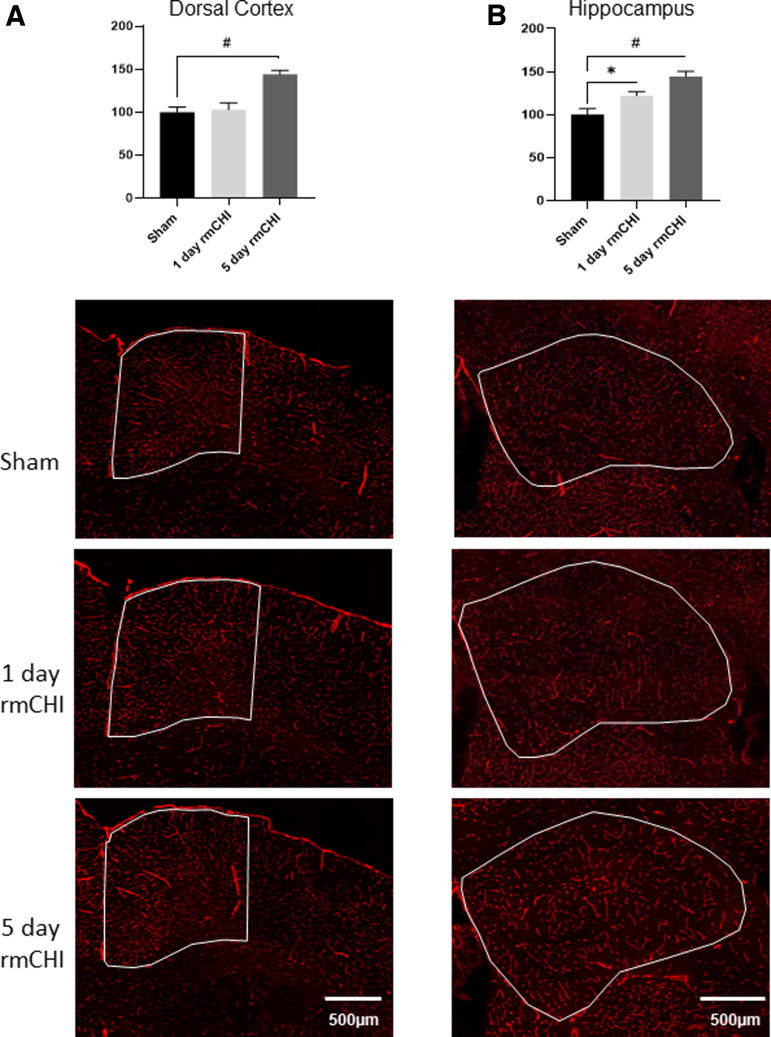
Immunolabeling of P-glycoprotein (Pgp) in sham, 1 day post-rmCHI, and 5 days post-rmCHI. **(A)** Dorsal cortex; **(B)** hippocampus. Overall effect is shown. *denotes p < 0.05; #denotes *p* < 0.01. Pgp, P-glycoprotein; rmCHI, repeat mild closed head injury.

Using the validated antibody, we examined the spatial changes in Pgp immunoreactivity following rmCHI. Figure 4A shows representative montaged images of Pgp immunoreactivity in the dorsal cortex of sham, 1-day post-rmCHI, and 5-day post-rmCHI mice. Pgp immunoreactivity was significantly increased by 5 days post-rmCHI in cortex proximal to the injury location (F_(2,13)_ = 14.37; *p* < 0.01). In the hippocampus (Fig. 4B), Pgp immunoreactivity on microvessels was significantly increased both 1 and 5 days after rmCHI (F_(2,13)_ = 13.79; *p* < 0.01).

### Effect on rmCHI on mRNA expression of BBB components

**FIG. 5.**
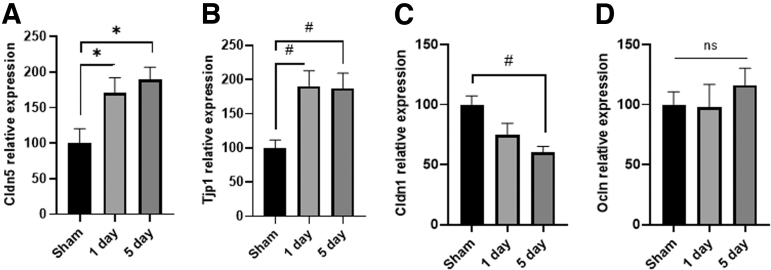
RT-PCR of BBB proteins in sham, 1 day post-rmCHI, and 5 days post-rmCHI. **(A)** Cldn1; **(B)** Cldn5; **(C)** Ocln; **(D)** Tjp1. Overall effect is shown. *denotes *p* < 0.05; #denotes *p* < 0.01. BBB, blood–brain barrier; Cldn1, claudin 1; Cldn5, claudin 5; rmCHI, repeat mild closed head injury; RT-PCR, real-time polymerase chain reaction; Tjp1, zona occludens.

Because Pgp is only responsible for substrate efflux out of the brain, we examined if rmCHI also elicited changes in the expression of proteins that make up the BBB, which regulates the influx of substances from circulation into the brain. The mRNA levels for the tight junction proteins claudin 5 (Cldn5; Fig. 5A; F_(2,12)_ = 5.65, *p* < 0.05), and zona occludens (Zo-1, also called Tjp1; Fig. 5B; F_(2,12)_ = 7.05, *p* < 0.01) were significantly increased at both 1 day and at 5 days post-injury. In contrast, mRNA expression for claudin 1 (Cldn1; Fig. 5C; F_(2,12)_ = 9.57, *p* < 0.01) decreased over time, and was significantly different by 5 days post-injury. No difference in the expression of occludin was detected at either time-point (Ocln; Fig. 5D; F_(2,12)_ = 0.45, n.s.).

## Discussion

ATP-binding cassette (ABC) transporters line the luminal side of the brain capillary endothelium and efflux substances, both neurotoxic and therapeutic, to prevent them from crossing the BBB and accessing the brain. An increase in transporter expression can lead to a decrease in the bioavailability of therapeutic drugs in the brain tissues. Of the ABC transporters, Pgp is the most active and has a broad range of substrate specificity, making it the transporter most associated with chemotherapeutic resistance.^7,10,23–26^ Using quantitative RT-PCR, we show that rmCHI increases the mRNA levels for both Pgp isoforms: Abcb1a and Abcb1b. Immunohistochemical analysis using a validated Pgp antibody indicated increased Pgp expression in the cortex proximal to the impact site as well as the hippocampus, a structure critical for learning and memory. Our results suggest that after rmCHI, any therapeutic agents that are Pgp substrates would likely be reduced in the hippocampus and dorsal cortex, and that inhibiting Pgp may be an effective strategy to increase bioavailability of these agents, potentially increasing their efficacy to reduce the pathophysiology of repeat concussive injury.

We determined that mRNA levels of both isoforms of mouse Pgp (Abcb1a and Abcb1b) are significantly increased as a result of rmCHI, beginning as early as 1 day after the last injury. In addition, the mRNA levels of BCRP (or Abcg2) trended upward after rmCHI. As Pgp has the broadest substrate specificity and Abcg2 shares the most extensive overlap of substrates with Pgp,^26–29^ increased expression of these genes suggests that a wide range of therapeutic drugs may be actively effluxed from the luminal membrane of the endothelial cells back into the blood stream, thereby reducing their efficacy.

Pgp expression has been extensively examined in the context of cancer and other diseases due to its prominent role in limiting the action of disease-modifying therapeutic drugs.^7,9,10,30–39^ Most of these studies have used commercial Pgp antibodies. Similar to that reported previously for other Pgp antibodies,^40–44^ our validation results using KO mice revealed that some tested antibodies had poor specificity, and caution must be taken when interpreting results of studies using these antibodies.

There are some caveats associated with the current work. The present study did not explore the mechanism(s) by which rmCHI increases Pgp mRNA and protein expression. At the transcription level, factors including NFkB and p53 have been shown to bind to the promoter region of Pgp.^45,46^ Signaling kinases that activate these transcription factors, such as the MAPK pathway and the PI3/Akt pathway, have been shown to increase Pgp expression.^47,48^ Although a number of studies have shown that experimental moderate-severe TBI activates these signaling pathways,^49–52^ it remains to be determined if rmCHI recruits these signaling mechanisms to increase Pgp mRNA. At the translational level, microRNAs (miRs) have also been shown to regulate Pgp protein expression level. For example, miR-145 has been shown to bind to the 3’-UTR of Pgp mRNA.^53^ Thus, altered expression of miRNA may also contribute to the regulation of Pgp protein expression following rmCHI. Finally, the group sizes evaluated were relatively small, which may have affected the statistical power. Additional studies will be required to confirm and extend these findings.

## Conclusion

Repeat concussive injury can exacerbate and prolong post-concussive syndrome (PCS).^54–61^ PCS can be classified into three categories: neurological (headache, disturbances in balance, vision changes, vomiting), cognitive (difficulty with concentration; problems with memory, decision making, and executive function), and behavioral (depression, insomnia, aggressive behavior). Several FDA-approved drugs (e.g., diazepam, tacrolimus, clozapine) are currently available to treat some of these symptoms. Unfortunately, a number of these drugs are also substrates for Pgp. As our data indicate that repeat concussive injury increases Pgp mRNA and protein expression, higher doses of these drugs may be needed to treat persons suffering from specific post-concussive symptoms (e.g., depression) resulting from repeat concussive injury. Alternatively, including a Pgp inhibitor (e.g., tariquidar, verapamil, ritanovir) with the treatment can also increase brain availability,^62^ and may limit unwanted side effects arising from high drug doses. Future studies could test this premise by treating rmCHI animals with a neuroprotective drug such as riluzole (a Pgp substrate) alone and in combination with a Pgp inhibitor to assess its relative effectiveness on improving outcome.

## Supplementary Material

Supplemental data

## Abbreviations Used

ABC: ATP-binding cassette
ALS: amyotrophic lateral sclerosis
ANOVA: analysis of variance
ATP: adenosine triphosphate
BBB: blood–brain barrier
BCA: bicinchoninic acid
BCRP: breast cancer resistance protein
BSA: bovine serum albumin
CCI: controlled cortical impact
cDNA: complementary DNA
Cldn1: claudin 1
Cldn5: claudin 5
CNS: central nervous system
C_T_: change in threshold cycle
ELISA: enzyme-linked immunosorbent assay
FDA: Food and Drug Administration
GFAP: glial fibrillary acidic protein
KO: knockout
miR: microRNA
mRNA: messenger RNA
mTBI: mild traumatic brain injury
Ocln: occludin
PBS: phosphate-buffered saline
PCR: polymerase chain reaction
PCS: post-concussive syndrome
Pgp: P-glycoprotein
rmCHI: repeat mild closed head injury
rmTBI: repeat mild traumatic brain injury
ROI: region of interest
RT-PCR: real-time polymerase chain reaction
SCI: spinal cord injury
SEM: standard error of the mean
Tjp1 or Zo-1: zona occludens
WT: wild type
