# Supplementary material for: P-glycoprotein Expression Is Upregulated in a Pre-Clinical Model of Traumatic Brain Injury
Source: Neurotrauma Rep. 2020 Nov 18;1(1):207–17. doi: 10.1089/neur.2020.0034 (PMC7703495; doi:10.1089/neur.2020.0034)
Supplement: Supplemental data [file Supp_Fig1.pdf]

Supplementary Data

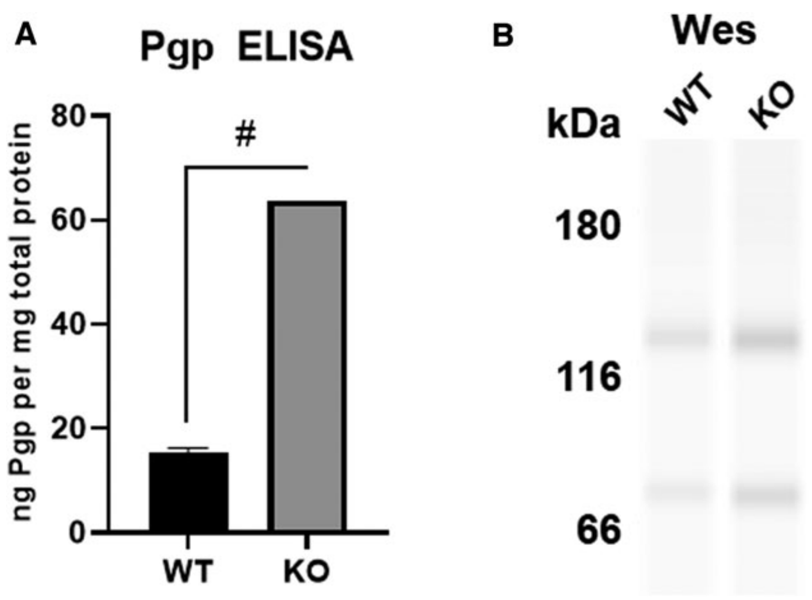

SUPPLEMENTARY FIG. S1. Validation of LSBio ELISA antibody. Equal amounts of KO and WT brain homogenate were tested by **(A)** ELISA, and a strong immunoreaction was detected in Mdr1a/b–/– mice. Similarly, when extracts from WT and null mice were run in a **(B)** capillary western blot, immunoreactive bands were detected in both brain tissues, suggesting that the anti-Mdr1 antibody supplied with the kit may be non-specific. ELISA, enzyme-linked immunosorbent assay; KO, knockout; Pgp, P-glycoprotein; WT, wild type.
